# Association of Obesity and Socioeconomic Status among Women of Childbearing Age Living in Urban Area of Morocco

**DOI:** 10.1155/2018/6043042

**Published:** 2018-07-29

**Authors:** Fatima Barich, Fatima Ezzahra Zahrou, Fatima Zahra Laamiri, Nisrine El Mir, Manelle Rjimati, Amina Barkat, El Arbi Rjimati, Hassan Aguenaou

**Affiliations:** ^1^Unité Mixte de Recherche en Nutrition et Alimentation URAC 39, Université Ibn Tofail-CNESTEN, RDC-Nutrition AFRA/AIEA, Kénitra, Morocco; ^2^Équipe de Recherche en Santé et Nutrition Du Couple Mère Enfant, FMP de Rabat, Université Mohammed V, Rabat, Morocco; ^3^Instituts Supérieurs des Professions Infirmières et Techniques de Santé, Rabat, Morocco

## Abstract

Worldwide, obesity is considered as an important public health problem. This study aims to explore the social and economic factors associated with overweight and obesity among women of childbearing age residing in the urban area of Morocco. This is a descriptive and analytical study conducted among women (*N*=240), aged between 15 and 49 years. At recruitment, socioeconomic status (SES) of each participant was assessed, anthropometric parameters were recorded, and body mass index (BMI), waist circumference, and waist-to-hip ratio (WHR) were measured to assess overweight and obesity. Data regarding skipped meals (breakfast, lunch, and dinner) were collected using an adapted questionnaire. The prevalence of overweight and obesity among women of childbearing age was 29.9% and 15.4%, respectively, while for abdominal obesity, the prevalence of overweight and obesity was, respectively, 39.9% and 60.1%. The results indicate that the prevalence of overweight and obesity among women is higher in women aged over 30. A significant association was shown between education level and both BMI and WHR (*r*_1_=−0.23, *r*_2_=−0.17, *p* < 0.05), respectively, and there is also a significant correlation between household size and WHR abdominal obesity (*r*=0.21, *p*=0.05). Our results reinforce the necessity to improve the access of all social classes in Morocco to reliable information on the determinants and consequences of obesity and to develop plans for adequate prevention and management of obesity.

## 1. Introduction

The worldwide prevalence of obesity has nearly tripled over the past 4 decades and thus has become a global epidemic that is still increasing in both developed and developing countries [1, 2]. In 2016, more than 1.9 billion adults, 18 years and older, were overweight [2]. Of these, over 650 million were obese and 39% of women aged 18 and over were overweight. Overweight and obesity are defined as abnormal or excessive fat accumulation that once developed into a disease may impair human health [3]. Together, they are considered as the fifth most common risk factor for death worldwide and account for at least 2.8 million deaths each year [3]. In addition, they substantially increase the risk of noncommunicable diseases, particularly cardiovascular diseases, endocrine and metabolic disturbances, non-insulin-dependent diabetes mellitus, several psychological problems, sleep apnea, osteoarthritis, and certain types of cancer [4, 5].

In Morocco, the prevalence of overweight and obesity is high, particularly in women from urban areas. In fact, the prevalence of overweight and obesity altogether accounted for 42% of women in urban regions and 29% in rural areas (National Survey on Population and Family Health) [6]. This prevalence exceeded 40% in the regions of Grand Casablanca, Oriental, Rabat-Salé-Zemmour-Zaër, and Fès-Boulmane (NSPF, 2003-2004). According to a national survey conducted among Moroccan women, more than 25% were overweight and 11% were obese (NSPF, 2003-2004) [6]. In 2012-2013, indicators showed that women and townspeople have seen their proportion of overweight increase from 29.9% to 34.7% and from 29.2% to 34.9% at the expense of their proportion of normal weight, which decreased from 50.6% to 36.1% and from 54.4% to 40.8%, respectively [7].

Numerous studies have shown a strong influence of socioeconomic status on obesity, particularly in women, causing variations in their behaviors which change their energy intake and energy expenditure and, as a result, affect their body fat storage [8–10]. Thus, this study aims to explore the social and economic factors associated with overweight and obesity among women of childbearing age residing in urban environments in Rabat and, as such, provides information that could help identify the groups most at risk for targeted interventions.

## 2. Methods

### 2.1. Study Design

This descriptive and analytical study conducted among women (*N*=240), aged between 15 and 49 years, lasted 3 months from April to June 2017.

This study included women who were aged between 15 and 49 years and living in the Rabat prefecture and not under any permanent medical treatment. Pregnant and lactating women, women suffering from mental illnesses, or those who participated in the pilot study were excluded from the present study.

The study received the approval of the Ethics Board of the Medicine and Pharmacy Faculty, University of Rabat, Morocco (Ethics no. 69 delivered on 31 January 2017). The purpose and the protocol of the study were presented and explained to the participants. Subsequently, oral and written consent was obtained from women, before the beginning of the survey.

### 2.2. Site of the Study

The study took place in Rabat which is located along the Atlantic Ocean at the Bouregreg River's outlet. Rabat, the capital of Morocco, is considered the second largest city with an urban population. This region is also known by its high obesity rate (NSPF, 2003-2004) [6].

### 2.3. Selection of Health Centers

Women were recruited from eight urban health centers that were selected on the basis of the following criteria: accessibility to our field team and large attendance of women enough to cover the required number for the study age range.

### 2.4. Sample

The study concerns 240 Moroccan women of childbearing age (15 to 49 years), who visited the health centers of Rabat between April and June 2017. Women had to meet the inclusion criteria and provide their consent to participate in the survey.

To calculate the sample size, we used the formula developed by Cochran [11] and Ardilly [12]:

(1) $$\begin{array}{c} n=t^{2}²\times p\times \frac{\left(1-p\right)}{m^{2}²}, \end{array}$$

where *t* is the 95% confidence level, *p* is the estimated prevalence of the obese population, and *m* is the margin of error (set at 5%).

The prevalence of obesity in the Rabat-Zemmour-Zaër region is estimated at 14.4% with a margin of error of 5% [6]. Thus, 189 subjects were considered necessary for inclusion to obtain statistically significant results. The total number of women who met the inclusion criteria was 240.

### 2.5. Data Collection

At recruitment, socioeconomic status (SES) of each participant was assessed, anthropometric parameters were recorded, and BMI, waist circumference, and WHR were measured to assess overweight and obesity. Data regarding skipped meals (breakfast, lunch, and dinner) were collected using an adapted questionnaire.

#### 2.5.1. Socioeconomic Questionnaire (SES)

The data on socioeconomic standards and living conditions of the women were collected at the beginning of the study by interviewing them. We used an adequate questionnaire that was adapted from other questionnaires used nationally to serve the purpose of our survey [6]. Information collected included the level of education of women, household size, number of children, marital status, household monthly global expenses, and alimentary expenses.

#### 2.5.2. Anthropometric Measurements

The anthropometric parameters of each participant were measured and were taken following the standard procedures used by the WHO [5].

Body weight was measured to the nearest 0.1 kg using a scale (seca 750) with minimal clothing and no shoes. Height was measured to the nearest 0.1 cm using a stadiometer (seca). BMI was calculated as a ratio of weight in kg and square height in m^2^. Women were classified into four groups according to BMI [5, 13]: underweight women with BMI less than 18.5 kg/m^2^; women in a normal range with a BMI between 18.5 kg/m^2^ and 24.99 kg/m^2^; overweight women with a BMI between 25 kg/m^2^ and 29.99 kg/m^2^; and obese women with a BMI greater than or equal to 30 kg/m^2^.

The waist circumference was measured at the approximate midpoint between the lower margin of the last palpable rib and the top of the iliac crest [14]. Hip circumference was measured at the widest point over the buttocks. For abdominal obesity, WHR was obtained by dividing the mean waist circumference by the mean hip circumference. Women with a WHR ≥ 0.85 were classified as obese [5].

### 2.6. Statistical Analysis

All statistical analyses were performed using the Statistical Package for the Social Sciences (SPSS, version 20.0). The distribution for normality of quantitative variables was tested using the Kolmogorov–Smirnov test. The variables normally distributed were presented as mean ± standard deviation (SD). The nominal variables were presented as proportion. The Spearman rank-order correlation was the test used for correlations to describe the linear relationship between two continuous variables. Two-sided *p* values < 0.05 were considered significant.

## 3. Results

The socioeconomic characteristics are presented in Table 1. The majority of women were Arabs, more than 50% do not work, and the prevalence of illiteracy was 13%. Families consist mainly of 4 to 7 persons. More than 40% of families live with more than 366 US$ per month, and 23% spend between 174.39 and 272.32 US$ monthly for food (Table 1).

The anthropometric parameters of participants are reported in Table 2. The mean age was 30.0 ± 9.9 years, with a mean weight of 64.0 ± 12.6 kg and an average height of 1.6 ± 0.1 cm. For body mass index (BMI), waist circumference (HC), hip circumference, and waist-to-hip ratio (WHR) in women, the average was 24.7 ± 4.8 kg/m^2^, 83.4 ± 12.9 cm, 99.4 ± 12.0 cm, and 0.8 ± 0.1 cm, respectively.

**Figure 1:**
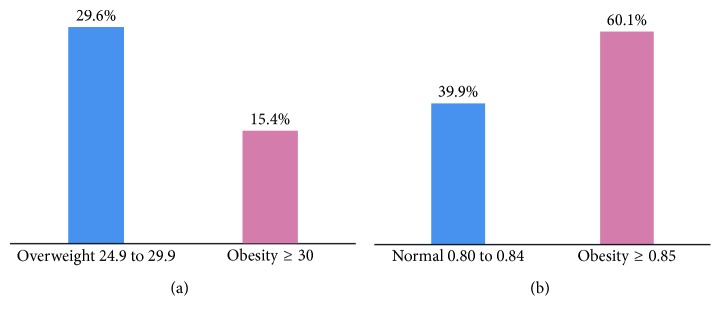
Overweight and obesity of women enrolled in the study. (a) BMI (kg/m^2^). (b) WHR (cm).

The classification of BMI and WHR is summarized in Figure 1. According to BMI, 29.6% of women were overweight and 15.4% were obese, while according to their WHR, almost 40% of women were not abdominally obese and 60.1% were abdominally obese (Figure 1).

Table 3 shows the anthropometric parameters of women of childbearing age by age strata. The results indicate that the mean of body mass index increased from the age of 30 (26.6 ± 4.3 kg/m^2^).

Table 4 shows the correlation between obesity and the socioeconomic status of participants. The analysis demonstrates the existence of a significant correlation between several variables. Indeed, the first correlation exists between age and BMI (*r*=0.43, *p* < 0.05) and WHR (*r*=0.25, *p* < 0.05). Another significant relationship was shown between education level with BMI and with WHR (*r*_1_=−0.23, *r*_2_=−0.17, *p* < 0.05), respectively. The same observation was found for marital status with BMI and with WHR (*r*=0.43, *r*_2_=0.15, *p* < 0.05). Finally, there is also a significant correlation between household size and WHR abdominal obesity (*r*=0.21, *p*=0.05).

Table 5 shows overweight and obesity among women skipping meals. Data analysis clearly demonstrated that 16.2% of women skipping breakfast were obese. However, more than 20% of women skipping dinner were overweight (Table 5). Indeed, more than 90% of women who were not skipping breakfast were overweight and 83.3% were obese.

## 4. Discussion

The study was designed to investigate the relationship between socioeconomic status, overweight, and obesity in Moroccan women. Our results showed that according to the body mass index, 29.6% of women were overweight and 15.4% were obese. These prevalences are considered higher than those reported in the 2003-2004 survey [6]. These results remain similar in the national studies [15, 16] and in other studies such as the meta-analysis carried out in the eastern Mediterranean region (EMRO) [17].

In our study, the prevalence of abdominal obesity is higher than the prevalence of overall obesity. Nowadays, excess weight is identified as an important risk factor for many noncommunicable diseases [18]. Indeed, several advances have been made in our understanding of the factors that contribute to obesity, such as the potential role of metabolic factors including the variations in energy expenditure and the identification of some genetic polymorphisms [19, 20]. According to our study, the prevalence of overweight and obesity among women is higher in women aged over 30. The authors from different countries previously published similar results. Indeed, similar observations were made in a prospective study conducted among Swedish women [21], in a study conducted in a Ghanaian population [22], and in a study in Nigeria [23].

On the contrary, several studies have highlighted the inverse association between socioeconomic status (SES) and risk of overweight and obesity. SES is commonly measured by the level of education, occupational status, and income [24, 25] corresponding to different potential individual mechanisms influencing lifestyle factors, such as having a healthy diet, regular physical activity, maintaining a healthy weight, and not smoking [26]. It would seem that married women are more susceptible to be overweight or obese. Marriage is a strong predictor that can contribute to women's obesity [28, 29]. This has been demonstrated by previous studies [15, 23, 27, 28]. In fact, this can be explained by the fact that fat is accumulated during subsequent pregnancies [29].

Education level is considered to influence obesity-related health behaviors, such as specific dietary pattern, physical exercise, smoking habits, and health- and nutrition-related knowledge and beliefs. Data analysis showed a negative correlation between education level and both general and abdominal obesity. These findings are consistent with the results of a survey conducted in 2011, which showed that the prevalence of obesity among adults with low levels of education is twice as high as that among adults with higher levels of education [7]. This can be explained by the fact that illiteracy or a low level of education seems to be another factor in obesity. Illiterate women are not aware of the consequences of obesity and the risk of unhealthy eating habits on health [29, 30]. These consequences are manifested in women of reproductive age in general by the risk of chronic disease [31]. At the reproductive level, overweight and obesity have been shown to be associated with negative reproductive health outcomes, thus reducing the efficacy of infertility treatment as well [31, 32].

These results are aligned with those from the study undertaken in 2009 proving that the fact of skipping meals disrupts the hormonal and digestive secretions, leading to an increase in energy “storage” and therefore to a weight increase [4]. Similarly, a study conducted among Swedish population showed that being obese was significantly associated with omitting breakfast (OR 1.41 (1.05–1.90)), omitting lunch (OR 1.31 (1.04–1.66)), and eating at night (OR 1.62 (1.10–2.39)) [33]. On the contrary, many observational studies have found that breakfast frequency is inversely associated with obesity and chronic disease [34]. Studies have suggested that eating breakfast everyday is associated with having a healthy body weight [35].

## 5. Conclusion

Our results reinforce the necessity to improve the access of all social classes in Morocco to reliable information on determinants and consequences of obesity and to develop plans for adequate prevention and management of obesity, taking into account the socioeconomic and environmental factors.

## Figures and Tables

**Table 1:** Socioeconomic characteristics of women of childbearing age.

Variables	*N*	(%)
*Age strata*		
15 to 19 yrs	54	22.50
20 to 29 yrs	66	27.50
30 to 39 yrs	74	30.83
40 to 49 yrs	46	19.17

*Culture*		
Arab	178	74.17
Amazigh	47	19.58
Sahrawi	7	2.92
Rifaine	6	2.50
Jebli	2	0.83

*Level of education*		
Illiterate	32	13.33
Primary	35	14.58
Secondary	109	45.42
Higher education	64	26.67

*Marital status*		
Single	87	36.25
Married	145	60.42
Divorced	7	2.92
Widowed	1	0.41

*Occupation of women*		
Without job	142	59.17
Student	52	21.67
With job	46	19 .16

*Occupation of the household head*		
Without job	8	3.33
With job	230	95.83
Retired	2	0.84

*Number of children*		
No children	92	38.33
1 to 2 children	93	38.75
3 and more	55	22.92

*Household size*		
<4 people	57	23.75
4 to 7 people	165	68.75
>7 people	18	7.50

*Housing type*		
Cement	229	95.42
Hard	11	4.58

*Monthly expenses*		
Lower than 122 US$	2	0.83
122 to 195 US$	12	5.00
196 to 244 US$	30	12.50
244 to 366 US$	28	11.67
Higher than 366 US$	99	41.25
Not known	69	28.75

*Monthly expenses for food*		
Lower than 65.36 US$	10	4.2
65.47 to 98.03 US$	12	5.0
98.14 to 130.71 US$	17	7.1
130.82 to 174.28 US$	51	21.3
174.39 to 272.32 US$	55	23.0
Higher than 272.32 US$	25	10.5
Not known	69	28.9

**Table 2:** Characteristics of anthropometric parameters of the studied population.

	*N*	Mean ± SD	Min	Max
Age (yrs)	240	30.0 ± 9.9	15	49
Weight (kg)	240	64.0 ± 12.6	39	105
Height (m)	240	1.6 ± 0.1	1.5	1.8
BMI (kg/m^2^)	240	24.7 ± 4.8	15	41
WC (cm)	240	83.4 ± 12.9	57	118
HC (cm)	240	99.4 ± 12.0	43	126
WHR (cm)	240	0.8 ± 0.1	0.6	1.5

*Note.* BMI: body mass index; WC: waist circumference; HC: hip circumference; WHR: waist-to-hip ratio.

**Table 3:** Anthropometric parameters of participants by age strata.

	*N*	Age	Weight	Height	BMI	WC	HC	WHR	*p*
15–19 yrs	54	17.4 ± 1.3	56.4 ± 10.4	1.6 ± 0.1	21.8 ± 3.7	74.8 ± 9.5	93.7 ± 11.0	0.80 ± 0.1	<0.0001
20–29 yrs	66	24.3 ± 2.6	61.7 ± 13.1	1.6 ± 0.1	23.5 ± 4.4	80.3 ± 12.4	97.1 ± 12.2	0.80 ± 0.1	<0.0001
30–39 yrs	74	35.4 ± 2.9	68.4 ± 11.3	1.6 ± 0.1	26.6 ± 4.3	88.1 ± 12.2	102.9 ± 12.4	0.90 ± 0.1	<0.0001
40–49 yrs	46	44.0 ± 3.0	69.2 ± 10.8	1.6 ± 0.1	27.1 ± 4.6	90.5 ± 11.3	103.9 ± 8.7	0.90 ± 0.1	<0.0001

*Note*. Results are presented as mean ± standard deviation. BMI: body mass index; WC: waist circumference; HC: hip circumference; WHR: waist-to-hip ratio. *p* value compared the anthropometric parameters by age strata using the ANOVA one-factor test.

**Table 4:** Association between anthropometric measures and socioeconomic status in studied women.

Variables		Weight	Height	BMI	WC	HC	WHR
		(*n*=240)	(*n*=240)	(*n*=240)	(*n*=240)	(*n*=240)	(*n*=240)
Age strata	*R*	0.39	−0.07	0.43	0.46	0.33	0.25
	*p* value	0.000^*∗*^	0.248	0.000^*∗*^	0.000^*∗*^	0.000^*∗*^	0.000^*∗*^

Culture	*R*	−0.02	0.00	−0.01	−0.05	0.01	−0.09
	*p* value	0.815	0.946	0.912	0.478	0.906	0.162

Level of education	*R*	−0.17	0.14	−0.23	−0.29	−0.19	−0.17
	*p* value	0.010^*∗*^	0.029^*∗*^	0.000^*∗*^	0.000^*∗*^	0.003^*∗*^	0.008^*∗*^

Marital status	*R*	0.38	−0.03	0.41	0.43	0.36	0.15
	*p* value	0.000^*∗*^	0.623	0.000^*∗*^	0.000^*∗*^	0.000^*∗*^	0.023^*∗*^

Occupation of women	*R*	−0.09	0.02	−0.10	−0.07	−0.08	−0.03
	*p* value	0.165	0.796	0.132	0.290	0.211	0.615

Occupation of the household head	*R*	−0.01	0.08	−0.04	−0.05	−0.04	−0.02
	*p* value	0.901	0.223	0.587	0.396	0.537	0.806

Number of children	*R*	0.43	−0.06	0.47	0.50	0.42	0.19
	*p* value	0.000^*∗*^	0.337	0.000^*∗*^	0.000^*∗*^	0.000^*∗*^	0.003^*∗*^

Household size	*R*	−0.11	−0.16	−0.06	0.03	−0.14	0.21
	*p* value	0.086	0.015^*∗*^	0.391	0.686	0.034^*∗*^	0.001^*∗*^

Housing type	R	0.02	−0.05	0.04	−0.02	−0.02	−0.02
	*p* value	0.734	0.437	0.572	0.764	0.803	0.723

Monthly expenses	*R*	−0.03	0.10	−0.07	−0.02	0.03	−0.08
	*p* value	0.608	0.130	0.285	0.804	0.659	0.206

Monthly expenses for food	*R*	−0.09	0.08	−0.12	−0.08	−0.03	−0.10
	*p* value	0.186	0.199	0.067	0.243	0.679	0.125

BMI: body mass index; WC: waist circumference; HC: hip circumference; WHR: waist-to-hip ratio; *r*: correlation coefficient. ^*∗*^The correlation is significant at the level of 0.05 (bilateral).

**Table 5:** Overweight and obesity among women who are (or are not) skipping meals.

	Breakfast		Lunch		Dinner	
	*N*	(%)	*N*	(%)	*N*	(%)
*Women skipping meals*						
Overweight	4	5.6	3	4.2	20	28.2
Obesity	6	16.2	1	2.7	5	13.5

*Women not skipping meals*						
Overweight	67	94.4	68	95.8	51	71.8
Obesity	31	83.8	36	97.3	32	86.5

^*∗*^Results are presented as proportion. Overweight: 24.9 to 29.9 kg/m^2^; obesity: 29.9 to 34.9 kg/m^2^.

## Data Availability

The data used to support the findings of this study are available from the corresponding author upon request.

## Abbreviations

SSE: Socioeconomic status
BMI: Body mass index
WC: Waist circumference
HC: Hip circumference
WHR: Waist-to-hip ratio
HCP: Haut Commissariat au Plan
SPSS: Statistical Package for the Social Sciences
EMRO: Eastern Mediterranean Regional Office.
